# Transcranial Magnetic Stimulation as a Therapeutic Option for Neurologic Diseases and Psychiatric Disorders: A Systematic Review

**DOI:** 10.7759/cureus.28259

**Published:** 2022-08-22

**Authors:** Huseyin Ozturk, Sathish Venugopal

**Affiliations:** 1 Neurology, California Institute of Behavioral Neurosciences & Psychology, Fairfield, USA

**Keywords:** transcranial magnetic stimulation, neuro-modulatory, non-invasive, psychiatric disorders, neurologic diseases

## Abstract

In the last two decades, transcranial magnetic stimulation (TMS) has attracted considerable interest in the research field and clinical applications because of its capacity to induce adequate electric current non-invasively for depolarizing cortex networks and superficial axons. Notably, the interest in TMS has been due to its ability to be utilized in exploring brain functioning. Indeed, reports have pointed out that TMS may effectively be used as a diagnostic and therapeutic approach for many neuropsychiatric diseases. However, they have not been sufficiently conclusive on the topic, with evidence showing mixed results. Against this backdrop, this systematic review explores TMS as a therapy option for neurologic diseases and psychiatric disorders. It summarizes and illustrates the current therapeutic uses of TMS in adults and children for detecting and treating neuropsychiatric conditions and prospective future applications. Using Preferred Reporting Items for Systematic Reviews and Meta-Analysis (PRISMA) 2020 guidelines, findings show that TMS is viable and has neuro-modulatory potential that can be employed successfully as a therapy alternative for neuropsychiatric disorders. Conversely, it is essential to more deeply understand the underlying mechanisms, alongside stimulation protocol optimization, of TMS for more practical applications.

## Introduction and background

Centuries ago, according to a myth, a shepherd named Magnes was wandering through part of Asia Minor, then known as Magnesia. Today, this place resides in southern Turkey. What he realized was his sandals stuck to the ground because of the attraction between the sandals’ iron nails and magnetite (lodestone). For a long time, people could not explain this force and tried to benefit from it. Hippocrates, referred to as the "Father of Medicine," claimed that lodestones had healing powers. Austrian Physician, Franz Anton Mesmer who began using magnetic lodestones in 1774 developed a theory concerning the body’s own “magnetic energy,” which he coined animal magnetism. There is also a famous painting in British Museum, “Patients undergoing magnetic therapy. Etching by J. Barlow, 1790, after J. Collings.” Until recent times magnets, magnetic fields, and their properties were not properly used as treatment. In 1985, magnets were used for the first time to stimulate and cure movement disorder [1].

Evidence has increasingly recognized non-invasive brain stimulation due to its potential as a therapeutic, diagnostic, and investigative tool. As a non-invasive technique, transcranial magnetic stimulation (TMS) or repetitive (rTMS) generates an electromagnetic flow throughout the brain by external pulsed magnetic fields near the material. Transcranial magnetic stimulation has been used successfully in children with psychiatric disorders and neurologic diseases to investigate normal and abnormal neurophysiology. Its potential as a treatment tool has been investigated. However, findings regarding its effectiveness in various neuropsychiatric disorders are inconclusive. Ironically, a once so-called quack theory that magnets healing properties come from their effects on iron in the blood and this effect leads to changes in blood flow is correct in some sense. Although TMS does not directly change blood flow, it changes the metabolism of neurons and so the blood flow to these neurons [2,3]. By using positron emission tomography (PET) records, it has been shown that TMS stimulation increases blood flow to stimulated brain areas by about 12% to 20% [4].

This systematic review aims to evaluate TMS as a therapeutic alternative for neurologic and psychiatric disorders. It briefly summarizes and demonstrates the latest treatment uses of TMS among children in identifying and providing therapy for these diseases, including its future applications.

## Review

Materials & methods

The evidence for the current systematic review followed Preferred Reporting Items for Systematic Reviews and Meta-Analysis (PRISMA) (2020) guidelines. This encompassed the series of steps detailed below.

Eligibility Criteria

The study included TMS intervention studies encompassing those with psychiatric disorders and neurologic diseases categorized by the WHO and published in English in scientific peer-reviewed journals for analysis. Besides, the studies were published from 2015 to 2022. Notably, reports, single-arm studies, case studies, and critical studies unpublished in peer-reviewed journals and dated earlier than the specified period were excluded.

Information Sources

Review articles published on the PubMed database were used as the source of information in this systematic review. As mentioned earlier, the articles should have been published from 2015 to 2022.

Search Strategy

A systematic screening was conducted for titles and abstracts of studies and review publications on the therapeutic uses of transcranial magnetic stimulation in movement disorders. The PubMed database was searched for peer-reviewed journals published from 2017 to the present to identify research for incorporation in this evaluation. Only human trials using TMS on patients with neurologic and psychiatric illnesses were selected. Additionally, the analyses examined the pertinent papers' reference lists. Key search terms included "psychiatric disease" OR “psychiatric disorder” OR “neurologic disease” OR “neurologic disorder” OR “amyotrophic lateral sclerosis” OR “cerebral palsy” OR “multiple sclerosis” OR “stroke” OR “spinal code injury” AND “transcranial magnetic stimulation” OR “repetitive transcranial magnetic stimulation” OR “minimally invasive brain stimulation” for intervention.

Selection Process

**Figure 1:**
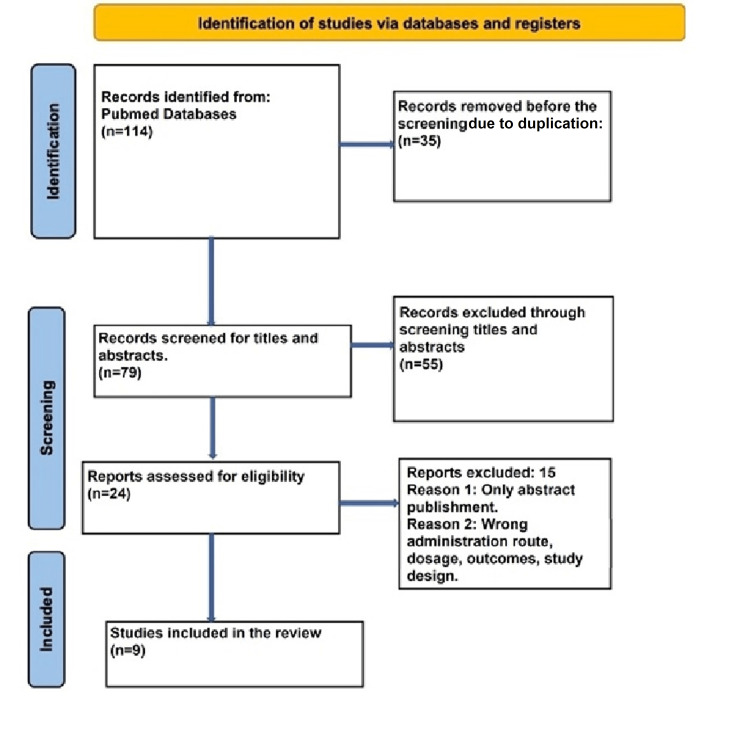
Flowchart of literature review search per Preferred Reporting Items for Systematic Reviews and Meta-Analysis (PRISMA 2020) guidelines

An online systematic review software was used for study selection performance. The whole set of generated studies from the search was independently reviewed, excluding those that did not meet the inclusion criteria and duplicates. Figure 1 presents the selection flowchart. For discrepancies, another reviewer was invited for a further evaluation.

Data Collection Process

The researcher reviewed the eligible evidence for inclusion and obtained the following data: study characteristics, including methods and relevant findings; TMS parameters; and outcomes.

Data Items and Effect Measures

The primary aim was to systematically review TMS as a therapeutic alternative for neurologic and psychiatric disorders. The effectiveness and efficacy of TMS were defined as a standardized mean change for pre and post-assessments, taking into account the severity of neuropsychiatric disease symptoms. The review focused on treatment and patient-level characteristics as future treatment change or response mediators at the individual patient level.

Study of Risk of Bias and Reporting Bias Assessment

Based on the review articles considered, a moderate risk of selection bias was noted to exist in the findings due to clinicians inevitably having to choose particular patient populations with specific neurological conditions and TMS treatment strategies. Most studies (67%) adequately detailed results. These findings seemed free of potential reporting bias.

Synthesis Methods

The statistical analyses were carried out using Microsoft Office 2019 (Microsoft Corporation, Redmond, WA) and Strata 14.1 (StataCorp. 2015. Stata Statistical Software: Release 14. College Station, TX: StataCorp LP). The researcher worked effortlessly in acquiring the randomized controlled trials (RCTs) and peer-reviewed journals meeting the inclusion criteria for extracting the data mentioned in the previous subsection. Furthermore, the data were analyzed, and any discrepancies were extensively discussed.

Certainty Assessment

Furthermore, the Cochrane Collaboration’s risk-of-bias guidelines were employed to evaluate the quality of the included evidence in this systematic review.

Results

Study Selection and Characteristics

Figure 1 indicates the election process used for choosing the studies. As shown, the search retrieved 114 studies; however, 79 pieces of evidence were screened against the abstract and title following the removal of duplicates. Of the 79 studies, 55 were excluded. This review ultimately examined 24 studies for full eligibility via full-text screening. From these, this systematic review excluded 15 articles because of only abstract publication and wrong administration route, dosage, outcomes, and study design. It finally included the remaining nine peer-reviewed article reviews for analysis. We assessed nine studies for quality appraisal using standardized quality assessment tools, and all articles were qualified after the quality appraisal.

Risk of Bias in Studies

From the included articles, it is imperative to point out that there was a low risk of bias for evidence associated with TMS as a therapeutic option for neurologic and mental diseases. All the studies were review studies according to the inclusion criteria. In all the studies, the outcomes were reported based on the effects of various neurologic and mental conditions. However, a good proportion of the evidence (up to 50%) did not sufficiently report as many diseases and disorders, including the possible ethical and safety issues of the novel therapeutic approach. Even though it was emphasized that participant allocation in the articles used in the reviews was conducted through a randomized sequence technique, the generation of a particular randomization sequence was not specified. Besides, most evidence (more than 70%) never determined how they attained allocation concealment. For this reason, the results were considered to contain a moderate-to-high selection bias risk. Less than 50% of the studies adequately disclosed data connected to follow-ups and drop-outs; contrarily, most articles (80%) gave detailed findings that were free of prospective reporting bias.

Results of Individual Studies

Table 1 summarizes the findings of each study. Authors, TMS methods, and relevant results have been included in the table. The extent and kinds of motor symptoms that improve vary among researchers. Similar to Latorre et al.’s study [5], Iglesias and Lefaucheur et al. also revealed encouraging evidence that TMS might enhance motor symptoms, obsessive-compulsive, and depression disorders in Parkinson’s disease [6,7]. However, its role in other movement disorders is unclear and has provided mixed results. Indeed, more recent evidence has indicated the presence of considerable data for considering TMS approval for various neurologic conditions, namely, dementia, migraine, spasticity, and stroke [8-10]. Like Narayana et al. [11], Habib et al. emphasize the TMS’ potential in diagnosing and treating children with neuropsychiatric disorders [12]. However, they contend that TMS may be associated with certain risks and safety issues. Finally, Somaa et al. found that TMS can be used in pioneering neuropsychiatric diseases such as consciousness disorders, multiple sclerosis, epilepsy, Alzheimer’s disease/mild cognitive impairment, and movement disorders [13].

**Table 1:** Summary of relevant studies TMS: transcranial magnetic stimulation

Study	Methods	Relevant findings
Latorre et al. [5]	The PubMed database searches and reviews articles	TMS might enhance motor symptoms and depression in Parkinson’s disease
Iglesias [6]	Literature review of data until 2018	TMS can improve life quality, motor impairment, depression, sclerosis, stroke, posttraumatic stress disorder
Lefaucheur et al. [7]	Article review/database search	TMS might enhance motor symptoms, obsessive-compulsive and depression disorders
Brandt et al. [8]	Article review/database search	rTMS can be applied in schizophrenia for the cognitive deficit, negative symptoms, and auditory hallucination treatment. However, other studies have shown its ineffectiveness in schizophrenia
Barker and Shields [9]	Article review/database search	TMS can effectively treat migraine. Besides, it is well-tolerated and safe by most individuals.
Burke et al. [10]	Article review	Besides being used for medication-resistant depression treatment, TMS is promising in other clinical applications, including Alzheimer’s disease and stroke.
Narayana et al. [11]	Article review/database search	TMS is a safe method used in children and adolescents for diagnosing and treating neurologic and psychiatric disorders for treatment and functional mapping.
Habib et al. [12]	Article review/database search	TMS can be used as a therapeutic and diagnostic device for neuropsychiatric diseases.
Somaa et al. [13]	Article review/database search	Clinical evidence shows that rTMS could be a viable therapeutic option for neurologic illnesses, namely consciousness disorders, multiple sclerosis, epilepsy, Alzheimer’s disease, and movement conditions.

Quality Assessment

We performed a thorough quality assessment for the nine confirmed articles using the National Heart, Lung, and Blood Institute (NHLBI) Study Quality Assessment Tools/Quality Assessment of Systematic Reviews and Meta-Analyses checklist (n=8).

Checklist scores of seven and above out of eight (for meta-analyses); six and above out of seven (for other than meta-analyses) were considered good quality articles. Out of nine articles, all scored good and were included in the review.

Discussion

Denouement

This study reviewed nine review articles focusing on the clinical applications of TMS individuals with neuropsychiatric disorders. The included evidence showed heterogeneity in specific areas of attention, used a similar methodological approach, and presented a moderate selection risk and reporting bias. Most papers suggest that TMS is a well-accepted, relatively safe, and painless neuro-electrophysiological method. This is consistent with other studies by Lu et al. [14], explaining that the approach may affect the cerebral cortex's neuro-electrical activity. This technique can also regulate the brain’s plasticity. Lu et al. reveal that TMS has been widely employed in treating neuropsychiatric disorders, including post-stroke rehabilitation.

Promising Implications

Overall, researchers have conducted the experiments in a restricted fashion due to a lack of definite understanding of TMS application and effects; by using different frequencies, outcomes could be observed and categorized into their respective types. For instance, Liu et al. contend that TMS can be categorized into low-frequency and high-frequency TMS, i.e., LF-TMS and HF-TMS [15]. Presently, theta-burst stimulation (TBS) has been used as a patterned TMS. Evidence suggests that LF-TMS on the non-affected side and continuous TBS may prevent cortical excitability; on the other hand, HF-TMS on the affected side and intermittent TBS enhance and hasten cortical excitability. Furthermore, many theories indicate that activating the contralateral hemisphere via HF stimulation fosters brain function re-organization and facilitates compensatory capacity in individuals with neuropsychiatric conditions. Such has been stated by Krogh et al. [16], as both Parkinson's patients and multiple sclerosis patients benefited from active rTMS sessions over contralateral to the affected side. The authors demonstrated TMS’s potential in improving patients with stroke by enhancing their day-to-day activities and upper limb movement. Nonetheless, it is essential to get pertinent evidence by directly comparing rather than systematically comparing various stimulation modalities.

Shortcomings of TMS

Despite the described benefits of TMS, TMS has been reported to have certain defects. Notably, because of the related supra-threshold peripheral signaling, the triple enhancement approach might cause some pain. Despite the robust and promising rationale for TMS use for treating neuropsychiatric illnesses, it can be unpleasant, which is the most prevalent adverse effect. Indeed, outcomes may not be as successful as anticipated [13]. Previous studies have similarly revealed this, stating that a lack of success is only observed in some neurologic and psychiatric conditions [9]. For instance, while TMS efficacy in motor symptoms treatment is reasonable, the same does not occur in major depression disorder.

Conflictions

Furthermore, the studies stressed that the variation across participants and patients is another TMS concern. Cash et al. suggest that enhancing TMS specificity through stimulation parameter changing can partially address this issue and can be achieved by using emerging tools like controllable TMS, permitting magnetic stimuli duration and shape change [17]. Moreover, despite showing great promises for the treatment of complex diseases such as schizophrenia [8] by the delivery of rTMS to the temporoparietal cortex and dorsolateral prefrontal cortex, and in major depressive disorder [18], to the left prefrontal cortex, the limited understanding of rTMS also impede TMS's success as a therapeutic device. Additionally, the present findings have limitations because of substantial non-reporting of the studies they included since the identified comparisons were not available for analyses, ultimately becoming a challenge in data syntheses. Accordingly, there could be over or under-estimation of the effectiveness and efficacy of TMS clinical applications in the current study.

## Conclusions

To summarize, TMS is viable and has neuro-modulatory potential that can be employed successfully as a therapy alternative for neuropsychiatric disorders. Conversely, it is essential to more deeply understand the underlying mechanisms, alongside a stimulation protocol optimization of TMS for more practical applications, due to the widely varying treatment effects and heterogeneity that the incorporated studies showed. Besides, bias may influence the results because of the significant non-reporting. For this reason, large RCTs should consider providing data to offer specific recommendations for eventual TMS application in neuropsychiatric therapy.
